# Enhancing COVID-19 Epidemic Forecasting Accuracy by Combining Real-time and Historical Data From Multiple Internet-Based Sources: Analysis of Social Media Data, Online News Articles, and Search Queries

**DOI:** 10.2196/35266

**Published:** 2022-06-16

**Authors:** Jingwei Li, Wei Huang, Choon Ling Sia, Zhuo Chen, Tailai Wu, Qingnan Wang

**Affiliations:** 1 School of Management Xi’an Jiaotong University Xi'an China; 2 Department of Information Systems City University of Hong Kong Hong Kong China; 3 National Center for Applied Mathematics Shenzhen Shenzhen China; 4 College of Business Southern University of Science and Technology Shenzhen China; 5 Department of Information Systems and Intelligent Business School of Management Xi’an Jiaotong University Xi'an China; 6 College of Public Health University of Georgia Athens, GA United States; 7 School of Economics University of Nottingham Ningbo China Ningbo China; 8 School of Medicine and Health Management Tongji Medical College Huazhong University of Science and Technology Wuhan China

**Keywords:** SARS-CoV-2, COVID 19, epidemic forecasting, disease surveillance, infectious disease epidemiology, social medial, online news, search query, autoregression model

## Abstract

**Background:**

The SARS-COV-2 virus and its variants pose extraordinary challenges for public health worldwide. Timely and accurate forecasting of the COVID-19 epidemic is key to sustaining interventions and policies and efficient resource allocation. Internet-based data sources have shown great potential to supplement traditional infectious disease surveillance, and the combination of different Internet-based data sources has shown greater power to enhance epidemic forecasting accuracy than using a single Internet-based data source. However, existing methods incorporating multiple Internet-based data sources only used real-time data from these sources as exogenous inputs but did not take all the historical data into account. Moreover, the predictive power of different Internet-based data sources in providing early warning for COVID-19 outbreaks has not been fully explored.

**Objective:**

The main aim of our study is to explore whether combining real-time and historical data from multiple Internet-based sources could improve the COVID-19 forecasting accuracy over the existing baseline models. A secondary aim is to explore the COVID-19 forecasting timeliness based on different Internet-based data sources.

**Methods:**

We first used core terms and symptom-related keyword-based methods to extract COVID-19–related Internet-based data from December 21, 2019, to February 29, 2020. The Internet-based data we explored included 90,493,912 online news articles, 37,401,900 microblogs, and all the Baidu search query data during that period. We then proposed an autoregressive model with exogenous inputs, incorporating real-time and historical data from multiple Internet-based sources. Our proposed model was compared with baseline models, and all the models were tested during the first wave of COVID-19 epidemics in Hubei province and the rest of mainland China separately. We also used lagged Pearson correlations for COVID-19 forecasting timeliness analysis.

**Results:**

Our proposed model achieved the highest accuracy in all 5 accuracy measures, compared with all the baseline models of both Hubei province and the rest of mainland China. In mainland China, except for Hubei, the COVID-19 epidemic forecasting accuracy differences between our proposed model (model i) and all the other baseline models were statistically significant (model 1, t_198_=–8.722, *P*<.001; model 2, t_198_=–5.000, *P*<.001, model 3, t_198_=–1.882, *P*=.06; model 4, t_198_=–4.644, *P*<.001; model 5, t_198_=–4.488, *P*<.001). In Hubei province, our proposed model's forecasting accuracy improved significantly compared with the baseline model using historical new confirmed COVID-19 case counts only (model 1, t_198_=–1.732, *P*=.09). Our results also showed that Internet-based sources could provide a 2- to 6-day earlier warning for COVID-19 outbreaks.

**Conclusions:**

Our approach incorporating real-time and historical data from multiple Internet-based sources could improve forecasting accuracy for epidemics of COVID-19 and its variants, which may help improve public health agencies' interventions and resource allocation in mitigating and controlling new waves of COVID-19 or other relevant epidemics.

## Introduction

COVID-19 poses extraordinary challenges for public health systems worldwide. As of November 26, 2021, COVID-19 had affected 222 countries and territories [1] and caused 259,502,031 confirmed cases, including 5,183,003 deaths worldwide [2]. Moreover, variants of the COVID-19 virus led to further challenges for public health. After the highly contagious Alpha variant swept across Europe and the United States in early 2021, the Delta variant replaced Alpha and became the dominant COVID variant worldwide [3]. The Delta variant is around 60% more transmissible than the Alpha variant, is moderately resistant to vaccines [4], and caused a new wave of the COVID-19 epidemic in Europe in late 2021 [5,6]. Omicron, an even more worrying variant, was reported from South Africa on November 24, 2021; it is said to out-compete the Delta variant and has been identified in Botswana, Belgium, Hong Kong, and Israel [7,8]. More timely and accurate forecasting of the incidence of COVID-19 and its variants is key to improving the efficiency of resource allocation and timeliness of intervention policy implementation [9-11].

Internet-based data sources, such as social media data (like microblogs), online news article data, and search query data, accumulate huge amounts of data all the time and have been proven to be an effective supplement to traditional infectious disease surveillance systems [12,13]. The underlying mechanism is that, before experiencing serious symptoms and going to a sentinel hospital, patients with symptoms may search for disease-related information on search engines like Google [14], complain about disease-related symptoms on social media like microblogs [15], or even share disease-related personal experiences on personal news articles platforms like instant articles [16]. This gives Internet-based data the ability to provide early warning for disease outbreaks [17,18] or provide supplemental information to enhance epidemic forecasting accuracy [14,16]. For instance, Wilson and Brownstein [19] retrieved official public health emergency–related online articles to support the early warning of Listeria outbreaks. Yang et al [14] proposed an autoregression model with Google search query data (AGRO) to improve the forecasting accuracy for influenza epidemics [14]. McGough et al [20] produced an improved estimation for the Zika virus in Latin America with a 1-week lead time. They used a multivariable linear regression model, combining real-time search query data, social media data (Twitter), outbreak news report counts, and historical officially reported case counts [20]. Internet-based data contain a large volume of unstructured text data [21] accompanied by noise caused by linguistic errors or misinformation [22]. To deal with Internet-based data, researchers have adopted a combination of methods, which include, but are not limited to, natural language processing, classification or clustering algorithms based on machine learning, and time-series models [12,23,24].

As COVID-19 has been and continues to be the most consequential infectious disease worldwide in this century, many researchers have used various Internet-based data sources to supplement COVID-19 surveillance [4,10,25]. Like previous research on other infectious diseases, COVID-19 forecasting research based on Internet-based data focuses mainly on 2 aspects: improving forecasting accuracy and improving forecasting timeliness. To improve COVID-19 forecasting accuracy, Shen et al [26] used the Granger causality test and showed that adding COVID-19 symptom–related microblogs could help enhance the COVID-19 predictive power. Liu et al [11] adopted a multivariable model and showed that adding real-time search query data and news article data into the traditional COVID-19 forecasting model could lead to more accurate forecasting results. The combination of different Internet-based data sources has shown greater power to enhance the forecasting accuracy of infectious diseases (including COVID-19) than using a single Internet-based data source [20]. However, existing methods incorporating more than one Internet-based data source used only real-time data from these sources as exogenous inputs but did not use historical data from all possible sources.

As for improving COVID-19 forecasting timeliness, Yuan et al [10] examined the lagged correlation between COVID-19 symptoms and core term–related search queries and daily new COVID-19 cases in the United States. They found that COVID-19–related search queries could provide a 12- to 14-day earlier warning for COVID-19 epidemics [10]. Similarly, Li et al [27] [26]proved that the Baidu search index and Weibo (social media platform similar to Twitter) index could both provide warning for COVID-19 outbreaks in China 8 days to 12 days earlier. However, the power of different Internet-based data sources to improve COVID-19 epidemic forecasting timeliness has not been fully explored [16]. The length of early warning time that Internet-based data could provide is not consistent across studies, varying from 0 [28] to 21 days [29]. Moreover, even though unofficial online news articles have shown great potential in supplementing COVID-19 surveillance [16,30,31], few studies have explored using unofficial online news articles to improve COVID-19 forecasting timeliness.

Our study explored whether combining real-time and historical data from multiple Internet-based sources could improve COVID-19 forecasting accuracy over the existing baseline models. We also compared COVID-19 forecasting timelines based on different Internet-based data sources.

## Methods

### Data Collection and Processing

We focused on the first wave of the COVID-19 epidemic in mainland China and compiled data on daily new confirmed COVID-19 case counts, online news articles, microblogs, and search queries from various sources. Following a previous study [26], we collected data from mainland China, with separate analyses for Hubei province and the remaining provinces. The official laboratory-confirmed case counts in mainland China, except Hubei province, can be retrieved since January 19, 2020 [21], while the official laboratory-confirmed case counts in Hubei province can be retrieved since January 10, 2020 [11]. The max time lags we explored were 20 days, following the example from previous studies [10,26]. Thus, we traced the Internet-based sources to December 21, 2019. We chose the end of our study period as February 29, 2020, when the primary wave of the COVID-19 epidemic in China had passed and the new confirmed case number decreased to single figures [21].

Daily new confirmed COVID-19 case counts were collected from the Chinese Center for Disease Control and Prevention (China CDC) website [32], which started collecting data on January 16, 2020. Earlier counts in Hubei province between January 10, 2020, and January 16, 2020, were compiled based on reports from the Health Commission of Hubei Province [33]. We then collected online news article data and microblog data from Sina Network Opinion Surveillance System (SNOSS) [34], a commercially available web-based platform that collects various Internet-based data in mainland China. Search query data were collected from the Baidu Index website [35]. We were the first to identify online news articles about COVID-19 and COVID-19–related microblogs using an approach based on COVID-19 core terms and symptom-related keywords. We also used COVID-19–related symptoms and core terms to extract COVID-19–related search queries, following a previous study [36]. Detailed Internet-based data extraction and filtering methods are described in Multimedia Appendix 1.

### Statistical Analysis

We first described the Internet-based data we retrieved and the COVID-19–related data we extracted. We then summarized all the COVID-19 forecasting-related data in 1 figure, including the fraction of online news articles and microblogs, search query counts, and lab-confirmed new case counts in mainland China, except Hubei, and Hubei province. All the data were normalized into an interval of 0 to 100 for better comparison. The figures aimed to show the Internet-based data sources’ potential to provide warnings for COVID-19 epidemics.

We also conducted lagged Pearson correlation analyses to evaluate the strength of relationships between different Internet-based data sources and daily new confirmed COVID-19 case counts. The max time lag explored was 20 days [26]. Because outliers can have a large influence on the Pearson correlation [37], we replaced the outlier data in Hubei on February 12, 2020, with the average of the 2 nearest neighbors [38]. A high correlation threshold of 0.7 was used, based on previous research [27].

### Model Formulation

Following previous infectious disease surveillance research [14,15,39], including COVID-19 forecasting research [11,26], we proposed an autoregressive model with exogenous inputs [40,41]. We used the proportion of daily new confirmed COVID-19 case counts as a dependent variable. For the proportions of daily new confirmed case counts bounded between 0 and 1, we used logit transformation on the variable to turn it into unbounded scores [14,39,42]. The proportion was calculated by dividing the number of new confirmed COVID-19 case counts over the related population, which was based on the latest Chinese national population census [43]. We then proposed our model by adding log-transformed COVID-19–related Internet-based data as exogenous inputs, including the fraction of online news article, microblogs, and search query counts. Let *p_t_* be the new confirmed COVID-19 case proportion. For days when *p_t_* = 0, we added a small positive number, *λ*, in the logit transformation. *λ* was calculated by dividing the square of the first quantile by the third quantile of all the proportions [44]. Let *y_t_* = *logit*(*p_t_*_+_ *λ*) be the logit-transformed new confirmed COVID-19 case proportion at day t. Let *x_t_* be the log-transformed fraction of COVID-19–related online news articles at day t, *z_t_* be the log-transformed fraction of COVID-19–related microblogs at day t, and *s_t_* be the log-transformed COVID-19–related search volume at day t. We chose “fever” to represent search queries, for it showed the highest correlations with new confirmed COVID-19 counts.

We proposed our autoregressive model with exogenous inputs, denoted as



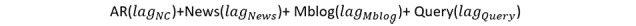



Incorporating the real-time and historical data from online news articles, microblogs, and search query volume:



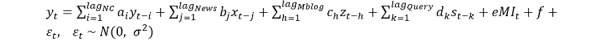



Where *a_i_* quantifies the contribution from the historical new confirmed COVID-19 case counts, *b_j_* quantifies the contribution from the historical fraction of COVID-19–related online news articles, *c_h_* quantifies the contribution from the historical fraction of COVID-19–related online news articles, *d_k_* quantifies the contribution from the historical COVID-19–related search queries, *M* is a binary variable that equals 1 when data are in Hubei and equals 0 when data are outside Hubei, *f* is a constant term, and *ɛ_t_* is a vector of independent random disturbance. *I_t_* is a time-varying binary variable that equals 1 on February 12, 2020, when Hubei adopted the fifth edition of the diagnostic criteria. *I_t_* controls for the exogenous shock of case counts on that day [26]. *lag_NC_*, *lag_News_*, *lag_Mblog_*, and *lag_Query_* ranged from 1 to 20 and were the optimal values that led to the highest forecasting accuracy (lowest root-mean-square error [RMSE]) for related baseline models described in the next paragraph using a single Internet-based data source (see Table S1 in Multimedia Appendix 2 for detailed lag selections).

We considered 5 baseline models, including (1) AR(*lag_NC_*): autoregression model based on historical new confirmed COVID-19 case counts only [16,26], (2) AR(*lag_NC_*)+News(*lag_News_*): autoregression model adding the fraction of COVID-19–related online news articles as an exogenous input [16], (3) AR(*lag_NC_*)+Mblog(*lag_Mblog_*): autoregression model adding the fraction of microblogs as an exogenous input [26], (4) AR(*lag_NC_*)+Query(*lag_Query_*): autoregression model adding search volume as an exogenous input [36], and (5) AR(*lag_NC_*)+News(1)+Mblog(1)+Query(1): multivariable linear model adding the fraction of real-time online news articles, the fraction of microblogs, and search query volume into historical official COVID-19 report data [11,20] (see Multimedia Appendix 3 for detailed model formulations).

Retrospective estimations of the daily proportion of confirmed COVID-19 counts were produced through the proposed model and baseline models. The estimation period was from January 19, 2020, to February 29, 2020, for mainland China, except for Hubei. For Hubei province, even though the official laboratory-confirmed COVID-19 cases can be retrieved since January 10, 2020, there was a severe lack of laboratory testing capacity at the beginning of this unexpected epidemic. Specifically, there were thousands of COVID-19–suspected cases that could not be confirmed due to the lack of testing capacity before January 27, 2020, and the daily test capacity in Hubei had to be extended 10 times on January 27, 2020 to address this issue [45]. The officially reported daily new confirmed COVID-19 case counts before January 27, 2020 reflected the testing capacity rather than the evolution of the epidemic. Thus, we tested the proposed model and other baseline models from January 27, 2020, to February 29, 2020, in Hubei.

We used the variance inflation factor (VIF) to measure multicollinearity in the independent variables. A VIF over 4 indicates a moderate level of multicollinearity, and a VIF exceeding 10 shows severe multicollinearity [46]. A repeated k-fold cross-validation [47,48] was adopted to evaluate the proposed model and baseline models. In this study, we split the data into 10 folds and repeated the cross-validation procedure 10 times [47]. We adopted the 5 most commonly used accuracy measures to compare the models’ forecasting results with the actual daily new confirmed COVID-19 case counts. The accuracy measures included the RMSE, mean absolute error (MAE), mean absolute percentage error (MAPE), correlation with forecasting target, and correlation of increment with forecasting target (the formulas for the accuracy indexes are presented in Multimedia Appendix 4) [14,49]. We conducted the analyses with the R version 4.0.2 statistical software package caret [50] version 6.0-86 and DAAG [51] version 1.24.

## Results

### Internet-Based Data Statistics

**Figure 1:**
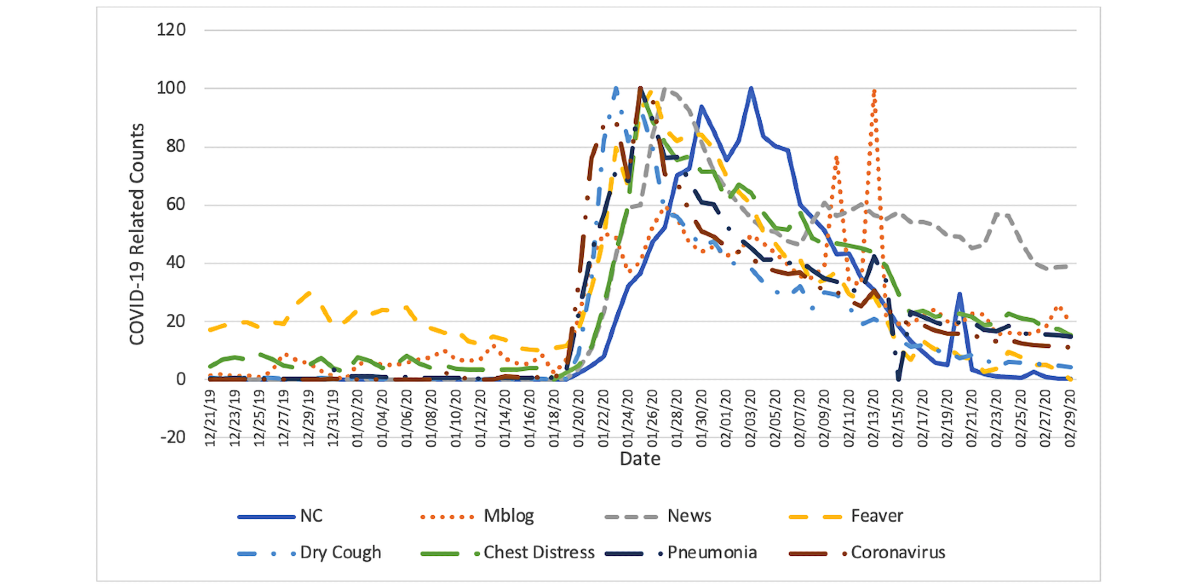
Daily time series of new confirmed COVID-19 case counts (NC), the fraction of COVID-19 related microblogs (Mblog), the fraction of COVID-19–related online news articles (News), and numbers of COVID-19–related search queries with the keyword “fever,” “dry cough,” “chest distress,” “pneumonia,” or “coronavirus” in mainland China, except Hubei province, from December 21, 2019 to February 29, 2020.

**Figure 2:**
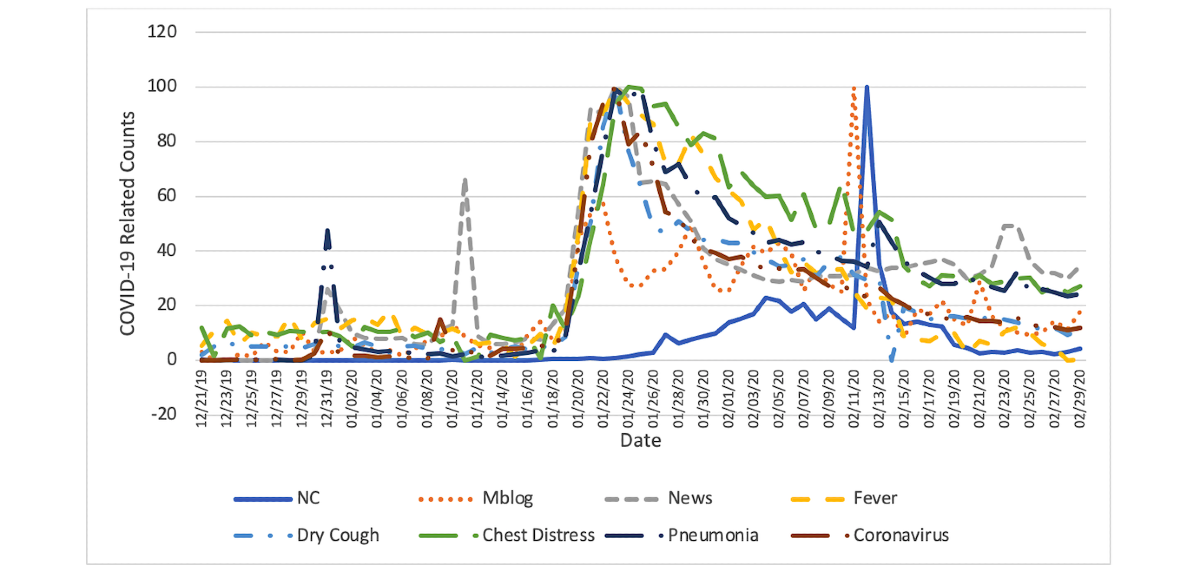
Daily time series of new confirmed COVID-19 case counts (NC), the fraction of COVID-19 related microblogs (Mblog), the fraction of COVID-19–related online news articles (News), and numbers of COVID-19–related search queries with the keyword “fever,” “dry cough,” “chest distress,” “pneumonia,” or “coronavirus” in Hubei province from December 21, 2019 to February 29, 2020.

Overall, we extracted 608,335 (out of 75,431,068) and 123,955 (out of 15,062,844) COVID-19–related online news articles for mainland China, except Hubei, and Hubei province separately, respectively. Unofficial online news articles accounted for about 92.8% (83,966,946/90,493,912) of all the news articles traced. We also identified 476,932 (out of 32,475,162) and 191,296 (out of 4,926,738) COVID-19–related microblogs posted in mainland China, except Hubei, and Hubei province, respectively. For the COVID-19–related search queries, we retrieved 24,165,139 queries in mainland China, except Hubei, and 988,402 related queries in Hubei province. The daily new confirmed COVID-19 case counts, the fraction of COVID-19–related online news articles, the fraction of COVID-19–related microblogs, and COVID-19–related search query counts are displayed in Figures 1 and 2.

Figure 1 shows that the first peak of daily confirmed COVID-19 case counts was reached on January 30, 2020, in provinces except Hubei. Compared with the official COVID-19 case counts, the peak in COVID-19–related online news articles was 2 days earlier (January 28, 2020), the peak in microblogs was 3 days earlier (January 27, 2020), and the peaks in search queries were 4 days to 7 days earlier (from January 23, 2020, to January 26, 2020).

Figure 2 shows that the highest peak of daily new confirmed COVID-19 case counts was reached on February 4, 2020, in Hubei province. Compared with the peak of official COVID-19 case counts, the peak in COVID-19–related online news articles was 12 days earlier (January 23, 2020), peak in microblogs was 13 days earlier (January 22, 2020), and peaks in search queries were 10 days to 12 days earlier (from January 23, 2020, to January 25, 2020). An outlier of incidence was found on February 12, 2020, when the new confirmed COVID-19 case counts increased dramatically as Hubei province started implementing the fifth edition of the COVID-19 diagnostic criteria. The new diagnostic criteria introduced more flexible diagnostic standards and turned many previously suspected cases into confirmed cases. This outlier could impact the forecasting accuracy and has been dealt with carefully in the model formulation and data analysis.

Lagged Pearson correlation analyses between different Internet-based data sources and daily new confirmed COVID-19 case counts were also conducted to illustrate the predictive power. The highest correlations for different sources with different time lags are summarized in Table 1 (see Tables S2 and S3 in Multimedia Appendix 2 for more details).

Table 1 shows that, in mainland China except Hubei, the highest correlation for online news articles was 0.619 with 2 days’ time lag, the highest correlation for microblogs was 0.613 with 2 days’ time lag, and the highest correlations for search queries ranged from 0.831 to 0.949 with time lags of 3 days to 6 days. In Hubei province, the highest correlation for online news articles was 0.667 with 14 days’ time lag, the highest correlation for microblogs was 0.632 with 7 days’ time lag, and the highest correlations for search queries ranged from 0.750 to 0.826 with time lags of 10 days to 12 days. Although the highest correlations for online news articles and microblogs were below the high correlation threshold (0.7), these correlations were all above 0.6, which was relatively high.

**Table 1:** Strongest correlation coefficients, *P* values, and related time lag between new confirmed COVID-19 case counts and the fraction of COVID-19–related microblogs, fraction of COVID-19–related online news articles, and numbers of COVID-19–related search queries between December 21, 2019, and February 29, 2020.

Source	Outside Hubei				Hubei		
	Highest correlation	*P* value	Days earlier	Highest correlation		*P* value	Days earlier
News articles	0.619	<.001	2	0.667		<.001	14
Microblogs	0.613	<.001	2	0.632		<.001	7
Search for “fever”	0.949	<.001	4	0.826		<.001	12
Search for “dry cough”	0.831	<.001	6	0.775		<.001	12
Search for “chest distress”	0.867	<.001	3	0.806		<.001	10
Search for “pneumonia”	0.854	<.001	5	0.750		<.001	11
Search for “coronavirus”	0.831	<.001	6	0.765		<.001	12

### Model Evaluation

The forecasting results for our proposed model and baseline models are presented in Tables 2 and 3. Optimal lags of different data sources, which result in the lowest RMSE for related baseline models incorporating a single Internet-based data source, are shown (see Table S1 in Multimedia Appendix 2 for the optimal lag selection). The last 2 columns show the paired *t* test results comparing our proposed model with the baseline models.

**Table 2:** COVID-19 epidemic forecasting model comparison for mainland China, except Hubei, between January 19, 2020, and February 29, 2020.

Model (lag)	Model number	RMSE^a^	MAE^b^	MAPE^c^	Correlation	Incremental correlation	t_198_	*P* value
AR(7)+News(1)+ Mblog(10)+Query(1)	model i	87.461	47.780	0.154	0.960	0.435	N/A^d^	N/A
AR(7)	model 1	152.182	97.852	0.579	0.852	0.006	–8.722	<.001
AR(7)+News(1)	model 2	117.223	68.158	0.374	0.911	0.066	–5.000	<.001
AR(7)+Mblog(10)	model 3	93.754	51.375	0.185	0.948	0.403	–1.882	.06
AR(7)+Query(1)	model 4	138.724	85.024	0.421	0.905	0.168	–4.644	<.001
AR(7)+News(1)+ Mblog(1)+Query(1)	model 5	90.494	53.332	0.306	0.954	0.167	–4.488	<.001

^a^RMSE: root-mean-square error.

^b^MAE: mean absolute error.

^c^MAPE: mean absolute percentage error.

^d^N/A: not applicable.

**Table 3:** COVID-19 epidemic forecasting model comparison for Hubei province, China, between January 27, 2020, and February 29, 2020.

Model (lag) (model no.)	Model number	RMSE^a^	MAE^b^	MAPE^c^	Correlation	Incremental correlation	t_198_	*P* value
AR(1)+News(3)+ Mblog(1)+Query(3)	model i	325.216	225.620	0.168	0.990	0.984	N/A^d^	N/A
AR(1)	model 1	658.238	403.665	0.267	0.963	0.958	–1.732	.09
AR(1)+News(2)	model 2	488.974	325.731	0.226	0.978	0.976	–1.196	.24
AR(1)+Mblog(1)	model 3	431.457	311.196	0.228	0.983	0.977	–0.252	.80
AR(1)+Query(3)	model 4	437.368	286.900	0.201	0.983	0.976	–0.364	.72
AR(1)+News(1)+ Mblog(1)+Query(1)	model 5	360.725	272.602	0.206	0.988	0.981	–0.965	.34

^a^RMSE: root-mean-square error.

^b^MAE: mean absolute error.

^c^MAPE: mean absolute percentage error.

^d^N/A: not applicable.

**Figure 3:**
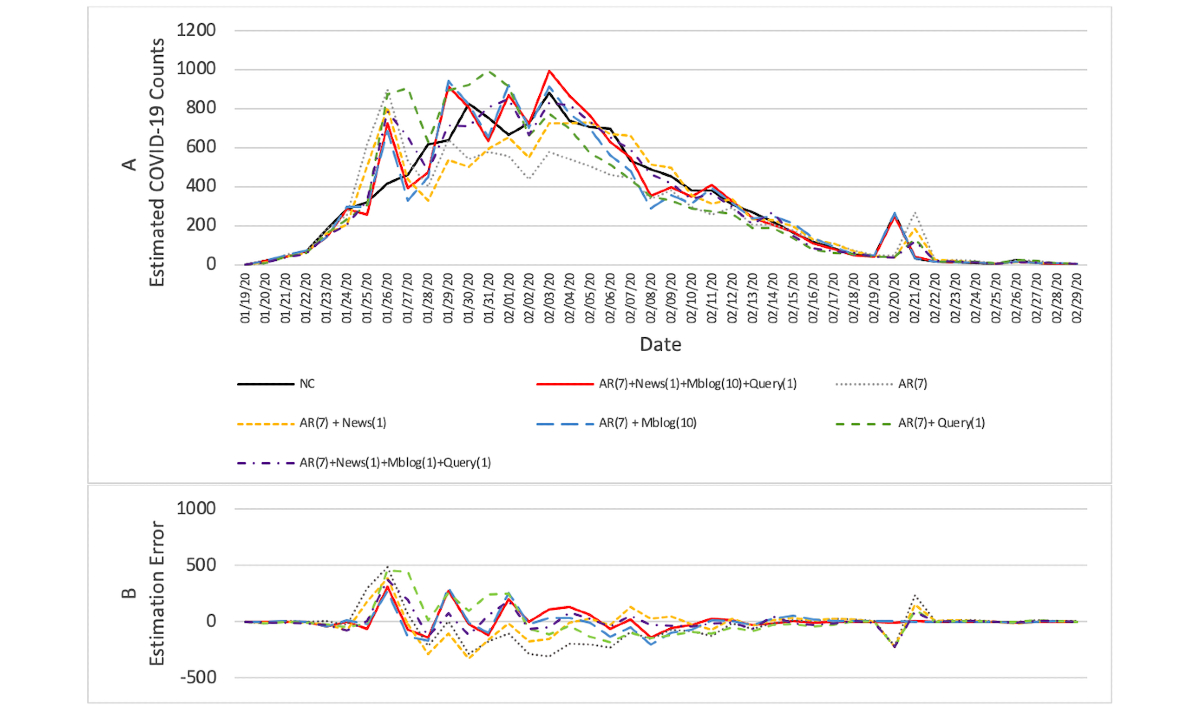
(A) Forecasting results for mainland China, except Hubei, between January 19, 2020 and February 29, 2020, during which the daily estimations of our proposed model and baseline models were compared against the daily new confirmed COVID-19 case counts (NC), and (B) the estimation error, defined as the estimated value minus the daily new confirmed COVID-19 case counts.

**Figure 4:**
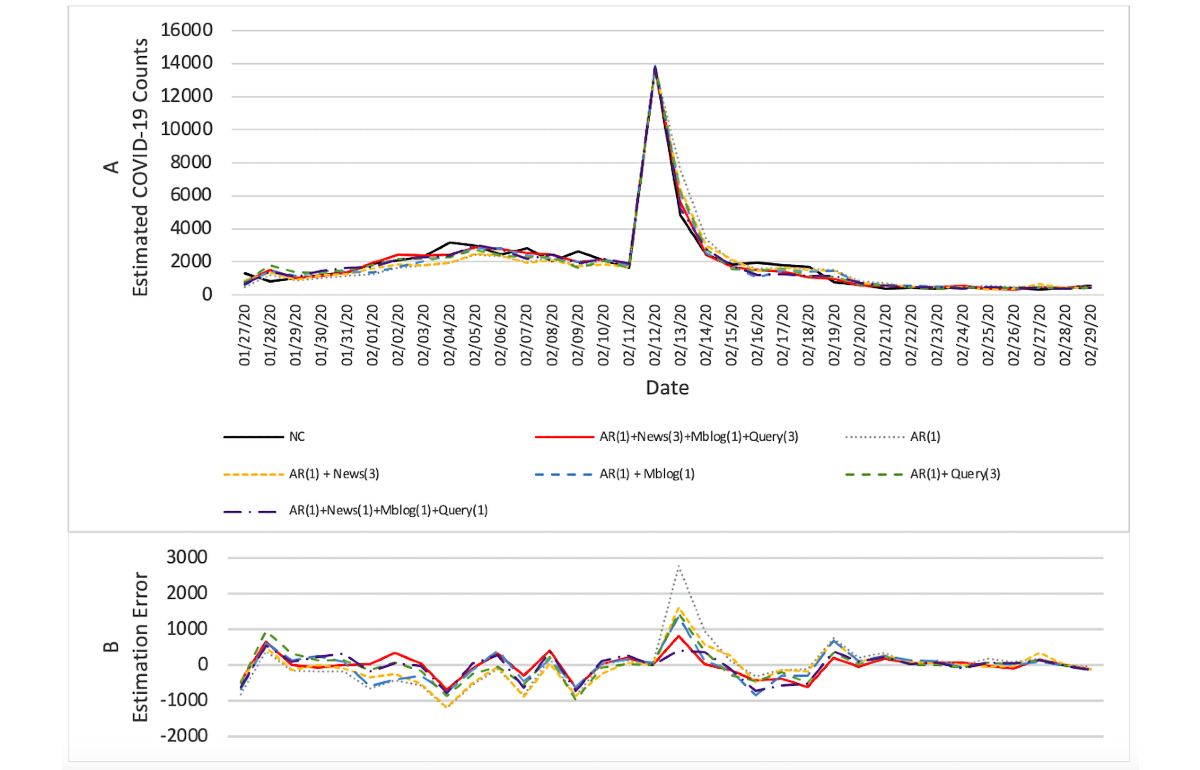
(A) Forecasting results for Hubei province between January 27, 2020 and February 29, 2020, during which the daily estimations of our proposed model and baseline models were compared against the daily new confirmed COVID-19 case counts (NC), and (B) the estimation error, defined as the estimated value minus the daily new confirmed COVID-19 case counts.

The results from the 5 accuracy measures were interpreted. The results in Tables 2 and 3 show that our proposed model (model i) achieved the highest accuracy in all 5 accuracy measures, compared with all the baseline models in both Hubei province and the rest of mainland China. Plots depicting forecasting results and estimation errors for the proposed model and baseline models are also shown in Figures 3 and 4.

We then assessed the statistical significance of the forecasting accuracy improvement between different models based on paired *t* tests on the models’ RMSEs. For mainland China, except Hubei, Table 2 and Figure 3 show that our proposed model (model i) could significantly improve the forecasting accuracy, compared with all the other baseline models (model 1, t_198_=–8.722, *P*<.001; model 2, t_198_=–5.000, *P*<.001; model 3, t_198_=–1.882, *P*=.06; model 4, t_198_=–4.644, *P*<.001; model 5, t_198_=–4.488, *P*<.001). For Hubei province, Table 3 and Figure 4 show our proposed model's (model i) forecasting accuracy improved significantly (at a significance level of .10) compared with the forecasting model using historical new confirmed COVID-19 case counts only (model 1, t_198_=–1.732, *P*=.09) and no significant differences compared with other baseline models (model 2, t_198_=–1.196, *P*=.24; model 3, t_198_=–0.252, *P*=.80; model 4, t_198_=–0.364, *P*=.72; model 5, t_198_=–0.965, *P*=.34). The forecasting accuracy differences between other baseline models using Internet-based data sources and model 1 are not significant (model 2, t_198_=–0.900, *P*=.37; model 3, t_198_=–1.630, *P*=.11; model 4, t_198_=–1.324, *P*=.19; model 5, t_198_=–0.786, *P*=.43).

We also evaluated the practical significance of the forecasting models from the perspective of MAPE. For provinces outside Hubei of mainland China in Table 2, our proposed model showed significant accuracy improvement. Specifically, our proposed forecasting model's unexplained error percentage was 15.4%, while the unexplained error percentages for the other models were as follows: forecasting model based on historical new confirmed COVID-19 case counts only (model 1), 57.9%; model incorporating COVID-19–related online news articles (model 2), 37.4%; model incorporating COVID-19–related microblogs (model 3), 18.5%; model incorporating COVID-19–related search queries (model 4), 42.1%; model combining real-time Internet-based sources into historical new COVID-19 case counts (model 5), 30.6%. Meanwhile, for Hubei province in Table 3, the improvement in accuracy with our proposed model was also nearly significant. The unexplained error percentage for our proposed model was 16.8%, while the unexplained error percentages for the other models were as follows: model 1, 26.7%; model 2, 22.6%; model 3, 22.8%; model 4, 20.1%; model 5, 20.6%.

The collinearity diagnostics revealed that real-time social media data, online news articles, and search queries are independent of each other in supplementing COVID-19 surveillance. More detailed results and discussions are presented in Multimedia Appendix 5.

## Discussion

### Principal Findings

The SARS-COV-2 virus and its variants pose extraordinary challenges for public health systems worldwide. More accurate forecasting of COVID-19 epidemics is key to improving the efficiency of resource allocation and the implementation of intervention policies [11,26]. Our proposed model innovatively incorporates both real-time and historical data from multiple Internet-based sources for COVID-19 epidemic forecasting. Tested during the first wave of the COVID-19 epidemic in mainland China, except Hubei, our proposed model showed statistically significant improved forecasting accuracy compared with the other baseline models. Tested in Hubei province, our proposed model outperformed all the baseline models in all 5 accuracy indexes, revealed significant practical influence, and showed statistically significant improved forecasting accuracy compared with baseline model 1 using the lab-confirmed case count only. Other baseline models incorporating different Internet-based data sources did not show significant differences compared with baseline model 1. This may be because people knew little of the disease at first and all talked online about the novel coronavirus pneumonia in Wuhan, Hubei, which could lead to disturbances in the Internet-based data sources [52]. In this condition, a single Internet-based data source or real-time data only may not be able to improve the COVID-19 forecasting accuracy, and our proposed model shows the ability to mitigate the disturbance and enhance COVID-19 surveillance by combining real-time and historical data from multiple Internet-based data sources.

This study also explored COVID-19 forecasting timeliness using different Internet-based data sources. Unlike previous studies that mainly focused on official online news articles, our study also took into account unofficial online news articles, which accounted for about 92.5% of all online news articles. The results show that COVID-19–related online news articles could provide a warning for the COVID-19 epidemic in mainland China, except Hubai, about 2 days earlier and in Hubai about 12 days to 14 days earlier. A similar early warning ability was also shown for microblogs and search queries. We found significant differences in the lag in an early warning for mainland China, except Hubei, and Hubei province, which may be caused by 2 reasons. First, Hubei experienced an extreme shortage of testing capacity in the beginning [26], which could have delayed the peak of lab-confirmed new case counts. Second, at the beginning of the first COVID-19 epidemic, people were curious about this unknown disease and tended to search or post related information even when they did not have associated symptoms [52]. This could advance the corresponding peak in Internet-based sources. As of the time of this writing, people were familiar with COVID-19–related information, and Internet-based sources, including online news articles, are supposed to provide a 2- to 6-day early warning for COVID-19 outbreaks.

Our study innovatively proposes core terms and symptom-related keyword-based approaches to extract COVID-19–related Internet-based data sources. The keyword-based approaches allow us to constantly and conveniently update the core terms and symptoms to keep up with the mutation of the COVID-19 virus. For example, people infected with the Delta variant are more likely to have a “runny nose,” “headache,” or “sore throat” and less likely to experience “loss of smell” [53]. Researchers then could focus more on the core term of “Delta variant” and the symptoms of “runny nose,” “headache,” and “sore throat” in online public data–based COVID-19 surveillance for this new round of epidemic in Europe [6]. We thus argue that our proposed model could help governments better prepare and respond to a new wave of COVID-19 and its variants.

Another interesting finding of our study is that the peak of daily new confirmed case counts in Hubei was reached on February 4, 2020, while the peak in the rest of mainland China was reached on January 30, 2020 (5 days earlier than Hubei Province). This finding was contrary to our common sense, for Hubei was the epicenter of the initial outbreak, and the rest of mainland China was influenced by this epidemic later. One possible reason for the delay of the COVID-19 epidemic peak in Hubei was the extreme shortage of medical resources at the beginning of the epidemic, including testing ability and hospital beds [26,45]. Many suspected cases could not be tested until the testing ability was extended 10 times on January 27 [45]. And until 15 mobile cabin hospitals were built in early February 2020, many confirmed cases with no or mild symptoms had to be quarantined at home rather than stay in the hospital, which increased the risk of COVID-19 transmission [54]. Different from Hubei, the rest of mainland China experienced a much smaller number of COVID-19 cases and had much more adequate medical resources [26], which made it possible to test and quarantine all the COVID-19 suspected cases in time. Thus, even though the rest of mainland China was influenced by the COVID-19 epidemic later than Hubei province, it is possible that the rest of mainland China could control the disease and reach the peak of daily new confirmed case counts earlier than Hubei. Future research could explore the factors contributing to the delay or advance of the epidemic peaks.

Overall, the results show that incorporating both real-time and historical data from multiple Internet-based sources into the COVID-19 forecasting model could significantly improve the forecasting accuracy, compared with other baseline models. Internet-based data sources, including online news articles, microblogs, and search queries, could provide early warning for COVID-19 outbreaks. These findings have broad public health implications. Internet-based data are timely, low-cost, and rich in information, making them critical in the surveillance of COVID-19 outbreaks. This application is even more important in rural areas, where the health infrastructure does not allow for widespread screening. COVID-19 surveillance using Internet-based data could provide much-needed information to help the government trace the outbreak and more effectively allocate resources, including testing capacity, oxygen cylinders, and hospital beds. Internet-based platforms allow users to capture detailed real-time snapshots of COVID-19–related events that happen to them or near them. As the COVID-19 virus continues to mutate, Internet-based sources with richer information have the potential to identify novel COVID-19 variants through deeper information analysis.

### Limitations

There are several limitations and potential future directions of this study that we would like to mention. First, our study only used retrospective data from mainland China and did not test the proposed model in countries that are currently experiencing an epidemic of COVID-19 and its variants. This is mainly because of data accessibility. We could not find available databases or online platforms that allowed us to access a large volume of real-time and historical microblogs and unofficial online news articles in other countries. We encourage future work to use the proposed method in different countries to test its generalizability and robustness.

Second, our study did not incorporate machine learning methods in the data filtering process. In this study, we explored the full database of Internet-based sources in mainland China from the SNOSS and Baidu Search Index, where the raw data are not available for downloading and further analysis. Future research could apply advanced machine learning methods to the raw data of various Internet-based sources to achieve more accurate epidemic-related data extraction and deeper information analyses. For example, future research can use the support vector machine to help extract COVID-19–related online data [55] or use a topic modeling algorithm to generate major themes about the COVID-19 epidemic [56]. Deeper content analyses could help identify real-time characteristics of the COVID-19 epidemic, which may act as early warning signals for new emerging COVID-19 variants or other epidemics.

Finally, our study mainly used symptom- and core term–related keywords to extract COVID-19–related Internet-based data, which has been proven to provide the most accurate predictions compared with other types of keywords [9,15]. Our underlying assumption is that, before getting severe symptoms and going to a sentinel hospital, patients with mild symptoms would likely search for or post COVID-19–related symptoms or core terms online. Our Internet-based method could identify patients with COVID-19 symptoms but lose sight of patients in the incubation period with no symptoms, which meant our method could only provide warning 2 days to 6 days earlier for the epidemic outbreaks. As our study’s major aim was to improve the COVID-19 forecasting accuracy, we did not explore new methods to improve the forecasting timeliness of Internet-based data in our study. We call for future studies to explore novel Internet-based sources, like traffic data and weather [21,57], to help improve the forecasting timeliness for COVID-19 epidemics.

### Conclusions

COVID-19 and its variants have been and continue to be a major public health threat worldwide. COVID-19 core term– and symptom-related Internet-based data could provide invaluable warning signals to the public and supplement existing COVID-19 surveillance systems. This study showed that our proposed COVID-19 forecasting method, incorporating both real-time and historical data from multiple Internet-based sources, could significantly improve the forecasting accuracy compared with other baseline models. Our results also show that Internet-based sources, including online news articles, could provide a warning 2 days to 6 days earlier for COVID-19 outbreaks.

## Appendix

Multimedia Appendix 1 Detailed descriptions of the Internet-based data extraction and filtering methods.

Multimedia Appendix 2 Supplementary tables.

Multimedia Appendix 3 Descriptions and formulations of baseline models.

Multimedia Appendix 4 Accuracy indexes.

Multimedia Appendix 5 Collinearity diagnostics.

## Abbreviations

CDC: Center for Disease Control and Prevention
MAE: mean absolute error
MAPE: mean absolute percentage error
RMSE: root-mean-squared error
SNOSS: Sina Network Opinion Surveillance System
VIF: variance inflation factor
