# Supplementary material for: Enhancing COVID-19 Epidemic Forecasting Accuracy by Combining Real-time and Historical Data From Multiple Internet-Based Sources: Analysis of Social Media Data, Online News Articles, and Search Queries
Source: JMIR Public Health Surveill. 2022 Jun 16;8(6):e35266. doi: 10.2196/35266 (PMC9205424; doi:10.2196/35266)
Supplement: Multimedia Appendix 1 [file publichealth_v8i6e35266_app1.docx]

## **Detailed Descriptions for Internet-based Data Extraction and Filtering Methods**

*COVID-19 Related Online News Articles*

We extracted COVID-19 related online news articles from SNOSS from December 21, 2019 (20 days before the first lab-confirmed reports in Hubei) to February 29, 2020. A previous study identified an official news article as infectious disease-related when the full text of the article contains at least one of the disease-related keywords [1]. However, in our case, massive official and unofficial online news articles mentioned the COVID-19 epidemic in Wuhan, Hubei, but many of them did not focus on the COVID-19. In this study, we used the headline with COVID-19 related core terms to help with extracting COVID-19 related online news articles. News headlines capture the essence of the articles and have been used to detect business events from massive online news articles [2]. Specifically, we extracted articles with headlines containing five COVID-19 related symptoms and core terms in Mandarin and one core term in English ("new coronavirus pneumonia" ["新型冠状病毒肺炎" in Mandarin], "novel coronavirus pneumonia" ["新冠肺炎"], "2019 coronavirus disease" ["2019冠状病毒病"], "pneumonia caused by new coronavirus" ["新型冠状病毒感染的肺炎"], "pneumonia of unknown etiology" ["不明原因肺炎"], and "COVID-19").

Unlike Microblog, in which users tend to share incidents around them [3], online news articles could publish articles referring to events in other areas [4]. We considered a news article covering a certain place only if the place’s name appeared most frequently in the news articles among all the places mentioned in the articles. We then used this location filtering method to extract official and unofficial online news articles referring to Hubei or other thirty provinces in mainland China. News articles on hot topics may be republished to attract site visits, leading to unstable estimates [5]. To deal with such noise, we filtered out articles more than 85% similar to previously published articles within 35 days. The fraction of online news articles was computed by dividing the COVID-19 related articles’ number over the overall retrieved articles’ number and served as an independent variable.

*COVID-19 Related Microblogs*

We extracted the COVID-19 related microblogs from SNOSS from December 21, 2019 (20 days before the first lab-confirmed reports in Hubei) to February 29, 2020. Symptom-related microblog posts have been proved to capture the trend of infectious diseases [6], including COVID-19 [7]. We summarized 25 COVID-19 related symptoms (see Table A1 below) based on guidelines from the World Health Organization [8], US CDC [9], and China CDC [10]. We first extracted microblogs containing at least one of these symptom-related keywords. Then, we adopted the method from Doan et al. [6] and filtered out the retweeted microblogs and microblogs with smiley emoticons, humor features, or URLs (see Table A2 below). The location tags, representing where the microblogs were posted, were used to distinguish microblogs in Hubei and outside Hubei. The fraction of microblogs was computed by dividing the COVID-19 related microblogs’ number over the overall retrieved microblogs’ number, and served as an independent variable.

*COVID-19 Related Search Query Data*

We extracted the COVID-19 related search queries from the Baidu Index platform [35] from December 21, 2019, to February 29, 2020. We identified two groups of keywords for the search query based on the literature review, including COVID-19 core term-related keywords and COVID-19 symptom-related keywords. Through literature review, we found Qin et al. (2020) provided a relatively comprehensive set of keywords for COVID-19 related search query extraction. Following Qin et al.’s work [11], we chose five keywords that were representative symptoms or core terms for COVID-19 in Mandarin ("fever" ["发烧"], "dry cough" ["干咳"], "chest distress" ["胸闷"], "coronavirus" ["冠状病毒"], and "pneumonia" ["肺炎"]). The location tags, that represent where the queries were searched, were used to distinguish microblogs in Hubei and provinces outside Hubei of mainland China.

## **Table A1.** COVID-19 Symptoms-related Keywords to Retrieve Microblogs (including Chinese keywords).

| Keywords | Translation | Keywords | Translation |
| --- | --- | --- | --- |
| Respiratory failure | 呼吸衰竭 | Sore throat | 咽痛 |
| Shortness of breath | 气促 | Headache | 头痛 |
| Loss of appetite | 食欲不振 | Muscle or joint pain | 肌痛 |
| Confusion | 意识混乱 | Different types of skin rash | 不同类型的皮疹 |
| Persistent pain in the chest | 持续胸痛 | Nausea | 恶心 |
| Persistent pressure in the chest | 持续胸闷 | Vomiting | 呕吐 |
| High temperature | 高烧 | Diarrhea | 腹泻 |
| Loss of taste | 失去味觉 | Chills | 发冷 |
| Loss of smell | 失去嗅觉 | Dizzy | 头晕 |
| Congestion | 鼻塞 | Fever | 发烧 |
| Runny nose | 流涕 | Dry cough | 干咳 |
| Conjunctivitis | 结膜炎 | Fatigue | 乏力 |
| Red eye | 红眼 |  |  |

## **Table A2.** Emoticon-, Humor- and URL-related Features to Help Filter Out Non-COVID19-related Microblogs (including Chinese Features).

| Emoticon features | :-) :) :D ^^ ^▽^ |
| --- | --- |
| **Humor features** | haha hhh hiahia hihi哈哈哈 吼吼 |
| **URL features** | http https ed2k Flashget thunder MMS mailto FTP |

## **Reference**

1. Kim J, Ahn I. Weekly ILI patient ratio change prediction using news articles with support vector machine. BMC bioinformatics. 2019;20(1):259. [doi: 10.1186/s12859-019-2894-2].

2. Papacharissi Z. The Importance of Being a Headline. 2018 [June 1, 2021]; 25-32]. Available from: https://thereader.mitpress.mit.edu/headlines-journalism/.

3. Lamb A, Paul M, Dredze M, editors. Separating fact from fear: Tracking flu infections on twitter. Proceedings of the 2013 Conference of the North American Chapter of the Association for Computational Linguistics: Human Language Technologies; 2013.

4. Li JL, Sia CL, Chen Z, Huang WW. Enhancing Influenza Epidemics Forecasting Accuracy in China with Both Official and Unofficial Online News Articles, 2019–2020. International Journal of Environmental Research and Public Health. 2021;18(12):6591.

5. Lazer D, Kennedy R, King G, Vespignani A. The parable of Google Flu: traps in big data analysis. Science. 2014;343(6176):1203-5. [doi: 10.1126/science.1248506].

6. Doan S, Ohno-Machado L, Collier N, editors. Enhancing Twitter data analysis with simple semantic filtering: Example in tracking influenza-like illnesses. 2012 iEEE second international conference on healthcare informatics, imaging and systems biology; 2012: IEEE.

7. Shen C, Chen A, Luo C, Zhang J, Feng B, Liao W. Using Reports of Symptoms and Diagnoses on Social Media to Predict COVID-19 Case Counts in Mainland China: Observational Infoveillance Study. Journal of Medical Internet Research. 2020;22(5):e19421. [doi: 10.2196/19421].

8. World Health Organization. Coronavirus disease (COVID-19). [Jun 11, 2021]; Available from: https://www.who.int/emergencies/diseases/novel-coronavirus-2019/question-and-answers-hub/q-a-detail/coronavirus-disease-covid-19#:~:text=symptoms.

9. Centers for Disease Control and Prevention. Symptoms of COVID-19. [Jun 11, 2021]; Available from: https://www.cdc.gov/coronavirus/2019-ncov/symptoms-testing/symptoms.html.

10. Chinese Center for Disease Control. 新型冠状病毒肺炎诊疗方案.

11. Qin L, Sun Q, Wang Y, Wu K-F, Chen M, Shia B-C, et al. Prediction of number of cases of 2019 novel coronavirus (COVID-19) using social media search index. International journal of environmental research and public health. 2020;17(7):2365. [doi: 10.3390/ijerph17072365].
