# Supplementary material for: Enhancing COVID-19 Epidemic Forecasting Accuracy by Combining Real-time and Historical Data From Multiple Internet-Based Sources: Analysis of Social Media Data, Online News Articles, and Search Queries
Source: JMIR Public Health Surveill. 2022 Jun 16;8(6):e35266. doi: 10.2196/35266 (PMC9205424; doi:10.2196/35266)
Supplement: Multimedia Appendix 2 [file publichealth_v8i6e35266_app2.docx]

## **Multimedia Appendix 2. Supplementary tables.**

## **Table S1.** Comparison of RMSE in Different Baseline Models with Different Time Lags: Hubei Province and the Rest of Mainland China.

| Model (lag) | RMSE (mainland China except Hubei) | Model (lag) | RMSE (Hubei Province) |
| --- | --- | --- | --- |
| AR(20) | 0.486 | AR(20) | 0.289 |
| AR(19) | 0.46 | AR(19) | 0.25 |
| AR(18) | 0.442 | AR(18) | 0.224 |
| AR(17) | 0.457 | AR(17) | 0.212 |
| AR(16) | 0.422 | AR(16) | 0.217 |
| AR(15) | 0.427 | AR(15) | 0.211 |
| AR(14) | 0.436 | AR(14) | 0.209 |
| AR(13) | 0.416 | AR(13) | 0.198 |
| AR(12) | 0.415 | AR(12) | 0.204 |
| AR(11) | 0.395 | AR(11) | 0.201 |
| AR(10) | 0.396 | AR(10) | 0.191 |
| AR(9) | 0.383 | AR(9) | 0.195 |
| AR(8) | 0.368 | AR(8) | 0.199 |
| **AR(7)** | **0.339** | AR(7) | 0.196 |
| AR(6) | 0.391 | AR(6) | 0.22 |
| AR(5) | 0.376 | AR(5) | 0.22 |
| AR(4) | 0.369 | AR(4) | 0.218 |
| AR(3) | 0.353 | AR(3) | 0.214 |
| AR(2) | 0.339 | AR(2) | 0.198 |
| AR(1) | 0.34 | **AR(1)** | **0.19** |
| AR(7)+News(20) | 0.831 | AR(1)+News(20) | 0.653 |
| AR(7)+News(19) | 0.841 | AR(1)+News(19) | 0.544 |
| AR(7)+News(18) | 0.774 | AR(1)+News(18) | 0.447 |
| AR(7)+News(17) | 0.719 | AR(1)+News(17) | 0.416 |
| AR(7)+News(16) | 0.6 | AR(1)+News(16) | 0.319 |
| AR(7)+News(15) | 0.737 | AR(1)+News(15) | 0.28 |
| AR(7)+News(14) | 0.753 | AR(1)+News(14) | 0.247 |
| AR(7)+News(13) | 0.671 | AR(1)+News(13) | 0.278 |
| AR(7)+News(12) | 0.613 | AR(1)+News(12) | 0.239 |
| AR(7)+News(11) | 0.607 | AR(1)+News(11) | 0.231 |
| AR(7)+News(10) | 0.597 | AR(1)+News(10) | 0.241 |
| AR(7)+News(9) | 0.62 | AR(1)+News(9) | 0.231 |
| AR(7)+News(8) | 0.553 | AR(1)+News(8) | 0.231 |
| AR(7)+News(7) | 0.462 | AR(1)+News(7) | 0.213 |
| AR(7)+News(6) | 0.387 | AR(1)+News(6) | 0.194 |
| AR(7)+News(5) | 0.375 | AR(1)+News(5) | 0.203 |
| AR(7)+News(4) | 0.327 | AR(1)+News(4) | 0.193 |
| AR(7)+News(3) | 0.325 | **AR(1)+News(3)** | **0.179** |
| AR(7)+News(2) | 0.306 | AR(1)+News(2) | 0.186 |
| **AR(7)+News(1)** | **0.273** | AR(1)+News(1) | 0.183 |
| AR(7)+Mblog(20) | 0.322 | AR(1)+Mblog(20) | 0.262 |
| AR(7)+Mblog(19) | 0.314 | AR(1)+Mblog(19) | 0.229 |
| AR(7)+Mblog(18) | 0.303 | AR(1)+Mblog(18) | 0.235 |
| AR(7)+Mblog(17) | 0.322 | AR(1)+Mblog(17) | 0.224 |
| AR(7)+Mblog(16) | 0.31 | AR(1)+Mblog(16) | 0.222 |
| AR(7)+Mblog(15) | 0.259 | AR(1)+Mblog(15) | 0.221 |
| AR(7)+Mblog(14) | 0.268 | AR(1)+Mblog(14) | 0.212 |
| AR(7)+Mblog(13) | 0.258 | AR(1)+Mblog(13) | 0.21 |
| AR(7)+Mblog(12) | 0.217 | AR(1)+Mblog(12) | 0.199 |
| AR(7)+Mblog(11) | 0.215 | AR(1)+Mblog(11) | 0.186 |
| **AR(7)+Mblog(10)** | **0.211** | AR(1)+Mblog(10) | 0.183 |
| AR(7)+Mblog(9) | 0.216 | AR(1)+Mblog(9) | 0.174 |
| AR(7)+Mblog(8) | 0.288 | AR(1)+Mblog(8) | 0.17 |
| AR(7)+Mblog(7) | 0.272 | AR(1)+Mblog(7) | 0.163 |
| AR(7)+Mblog(6) | 0.369 | AR(1)+Mblog(6) | 0.163 |
| AR(7)+Mblog(5) | 0.368 | AR(1)+Mblog(5) | 0.162 |
| AR(7)+Mblog(4) | 0.354 | AR(1)+Mblog(4) | 0.174 |
| AR(7)+Mblog(3) | 0.333 | AR(1)+Mblog(3) | 0.165 |
| AR(7)+Mblog(2) | 0.337 | AR(1)+Mblog(2) | 0.163 |
| AR(7)+Mblog(1) | 0.346 | **AR(1)+Mblog(1)** | **0.16** |
| AR(7)+Query(20) | 0.463 | AR(1)+Query(20) | 0.267 |
| AR(7)+Query(19) | 0.451 | AR(1)+Query(19) | 0.243 |
| AR(7)+Query(18) | 0.374 | AR(1)+Query(18) | 0.22 |
| AR(7)+Query(17) | 0.385 | AR(1)+Query(17) | 0.231 |
| AR(7)+Query(16) | 0.387 | AR(1)+Query(16) | 0.206 |
| AR(7)+Query(15) | 0.381 | AR(1)+Query(15) | 0.195 |
| AR(7)+Query(14) | 0.369 | AR(1)+Query(14) | 0.211 |
| AR(7)+Query(13) | 0.348 | AR(1)+Query(13) | 0.199 |
| AR(7)+Query(12) | 0.33 | AR(1)+Query(12) | 0.197 |
| AR(7)+Query(11) | 0.326 | AR(1)+Query(11) | 0.184 |
| AR(7)+Query(10) | 0.319 | AR(1)+Query(10) | 0.185 |
| AR(7)+Query(9) | 0.307 | AR(1)+Query(9) | 0.199 |
| AR(7)+Query(8) | 0.295 | AR(1)+Query(8) | 0.204 |
| AR(7)+Query(7) | 0.273 | AR(1)+Query(7) | 0.184 |
| AR(7)+Query(6) | 0.277 | AR(1)+Query(6) | 0.175 |
| AR(7)+Query(5) | 0.31 | AR(1)+Query(5) | 0.17 |
| AR(7)+Query(4) | 0.34 | AR(1)+Query(4) | 0.17 |
| AR(7)+Query(3) | 0.305 | **AR(1)+Query(3)** | **0.161** |
| AR(7)+Query(2) | 0.281 | AR(1)+Query(2) | 0.168 |
| **AR(7)+Query(1)** | **0.267** | AR(1)+Query(1) | 0.166 |

*Boldface highlights the lowest RMSE with optimal lag value for each baseline model.

## **Table S2.** Correlation Coefficients between Daily New Lab-confirmed COVID-19 Case Counts and Lagged Fraction of COVID-19 Related Microblogs, Fraction of COVID-19 Related Online News Articles, and COVID-19 Related Search Query Count: Mainland China except Hubei, December 21, 2019- February 29, 2020.

| Days earlier | Microblog | News Articles | Fever | Dry cough | Chest Distress | Pneumonia | Coronavirus |
| --- | --- | --- | --- | --- | --- | --- | --- |
| 0 | .549^**^ | .568^**^ | .723^**^ | .429^**^ | .800^**^ | .569^**^ | .390^*^ |
| 1 | .591^**^ | .608^**^ | .815^**^ | .545^**^ | .847^**^ | .675^**^ | .513^**^ |
| 2 | **.613^**^** | **.619^**^** | .882^**^ | .645^**^ | .862^**^ | .764^**^ | .630^**^ |
| 3 | .606^**^ | .596^**^ | .933^**^ | .724^**^ | **.867^**^** | .832^**^ | .724^**^ |
| 4 | .562^**^ | .552^**^ | **.949^**^** | .781^**^ | .833^**^ | .860^**^ | .785^**^ |
| 5 | .509^**^ | .493^**^ | .944^**^ | .825^**^ | .785^**^ | **.854^**^** | .830^**^ |
| 6 | .461^**^ | .434^**^ | .899^**^ | **.831^**^** | .697^**^ | .843^**^ | **.831^**^** |
| 7 | .486^**^ | .362^*^ | .849^**^ | .808^**^ | .591^**^ | .795^**^ | .827^**^ |
| 8 | .350^*^ | .257 | .765^**^ | .765^**^ | .483^**^ | .720^**^ | .799^**^ |
| 9 | .252 | .139 | .650^**^ | .683^**^ | .369^*^ | .628^**^ | .728^**^ |
| 10 | .173 | .036 | .528^**^ | .584^**^ | .228 | .503^**^ | .611^**^ |
| 11 | .038 | -.070 | .407^**^ | .503^**^ | .098 | .382^*^ | .507^**^ |
| 12 | -.062 | -.176 | .256 | .387^*^ | -.039 | .257 | .398^**^ |
| 13 | -.144 | -.270 | .109 | .252 | -.172 | .128 | .280 |
| 14 | -.241 | -.353^*^ | -.041 | .116 | -.292 | .002 | .150 |
| 15 | -.335^*^ | -.423^**^ | -.188 | -.016 | -.390^*^ | -.148 | .016 |
| 16 | -.419^**^ | -.484^**^ | -.304 | -.131 | -.466^**^ | -.251 | -.101 |
| 17 | -.454^**^ | -.535^**^ | -.395^**^ | -.222 | -.519^**^ | -.331^*^ | -.204 |
| 18 | -.513^**^ | -.573^**^ | -.476^**^ | -.307^*^ | -.563^**^ | -.414^**^ | -.299 |
| 19 | -.550^**^ | -.605^**^ | -.532^**^ | -.372^*^ | -.604^**^ | -.480^**^ | -.372^*^ |
| 20 | -.560^**^ | -.627^**^ | -.577^**^ | -.426^**^ | -.630^**^ | -.532^**^ | -.436^**^ |

*Boldface highlights the highest Pearson correlations for different Internet-based data sources with different time lags.

† ** means the correlation is significant at the 0.01 level (2-tailed), and * means the correlation is significant at the 0.05 level (2-tailed).

## **Table S3.** Correlation Coefficients between Daily New Lab-confirmed COVID-19 Case Counts and Lagged Fraction of COVID-19 Related Microblogs, Fraction of COVID-19 Related Online News Articles, and COVID-19 Related Search Query Count: Hubei Province, December 21, 2019- February 29, 2020.

| Days earlier | Microblog | News Articles | Fever | Dry cough | Chest Distress | Pneumonia | Coronavirus |
| --- | --- | --- | --- | --- | --- | --- | --- |
| 0 | .235 | -.135 | .057 | .137 | .342^*^ | .218 | .029 |
| 1 | .424^**^ | -.082 | .133 | .210 | .398^**^ | .264 | .086 |
| 2 | .579^**^ | -.030 | .238 | .297^*^ | .501^**^ | .346^*^ | .168 |
| 3 | .441^**^ | .054 | .346^*^ | .389^**^ | .590^**^ | .418^**^ | .243 |
| 4 | .473^**^ | .135 | .437^**^ | .463^**^ | .638^**^ | .497^**^ | .331^*^ |
| 5 | .539^**^ | .213 | .516^**^ | .530^**^ | .688^**^ | .554^**^ | .411^**^ |
| 6 | .577^**^ | .304^*^ | .591^**^ | .580^**^ | .731^**^ | .602^**^ | .485^**^ |
| 7 | **.632^**^** | .373^**^ | .636^**^ | .625^**^ | .756^**^ | .654^**^ | .545^**^ |
| 8 | .571^**^ | .433^**^ | .694^**^ | .648^**^ | .786^**^ | .686^**^ | .593^**^ |
| 9 | .572^**^ | .494^**^ | .750^**^ | .691^**^ | .803^**^ | .726^**^ | .656^**^ |
| 10 | .565^**^ | .540^**^ | .783^**^ | .732^**^ | **.806^**^** | .742^**^ | .715^**^ |
| 11 | .545^**^ | .606^**^ | .819^**^ | .762^**^ | .791^**^ | **.750^**^** | .747^**^ |
| 12 | .513^**^ | .648^**^ | **.826^**^** | **.775^**^** | .753^**^ | .745^**^ | **.765^**^** |
| 13 | .509^**^ | .654^**^ | .812^**^ | .753^**^ | .721^**^ | .716^**^ | .756^**^ |
| 14 | .519^**^ | **.667^**^** | .794^**^ | .689^**^ | .652^**^ | .655^**^ | .718^**^ |
| 15 | .496^**^ | .643^**^ | .729^**^ | .636^**^ | .569^**^ | .611^**^ | .676^**^ |
| 16 | .397^**^ | .639^**^ | .621^**^ | .554^**^ | .510^**^ | .548^**^ | .608^**^ |
| 17 | .320^*^ | .588^**^ | .550^**^ | .474^**^ | .465^**^ | .489^**^ | .562^**^ |
| 18 | .291^*^ | .533^**^ | .490^**^ | .423^**^ | .398^**^ | .458^**^ | .548^**^ |
| 19 | .317^*^ | .514^**^ | .431^**^ | .391^**^ | .350^*^ | .428^**^ | .507^**^ |
| 20 | .266 | .535^**^ | .372^**^ | .404^**^ | .303^*^ | .379^**^ | .469^**^ |

*Boldface highlights the highest Pearson correlations for different Internet-based data sources with different time lags.

† ** means correlation is significant at the 0.01 level (2-tailed), and * means correlation is significant at the 0.05 level (2-tailed).
