# Supplementary material for: Enhancing COVID-19 Epidemic Forecasting Accuracy by Combining Real-time and Historical Data From Multiple Internet-Based Sources: Analysis of Social Media Data, Online News Articles, and Search Queries
Source: JMIR Public Health Surveill. 2022 Jun 16;8(6):e35266. doi: 10.2196/35266 (PMC9205424; doi:10.2196/35266)
Supplement: Multimedia Appendix 3 [file publichealth_v8i6e35266_app3.docx]

## **Multimedia Appendix 3. Descriptions and formulations of baseline models.**

1. Autoregression model based on historical COVID-19 new confirmed case counts only [16,26], denoted as AR(${lag}_{NC}$):

$y_{t}=\sum_{i=1}^{{lag}_{NC}} a_{i}y_{t-i}+eMI_{t}+f+\varepsilon_{t},$ $\varepsilon_{t} \sim N(0, \sigma^{2})$ (1)

1. Autoregression model adding the fraction of COVID-19 related online news articles as exogenous input [16], denoted as AR(${lag}_{NC}$)+News(${lag}_{News}$):

$y_{t}=\sum_{i=1}^{{lag}_{NC}} a_{i}y_{t-i}+\sum_{j=1}^{{lag}_{News}} b_{j}x_{t-j}+eMI_{t}+f+\varepsilon_{t},$ $\varepsilon_{t} \sim N(0, \sigma^{2})$ (2)

1. Autoregression model adding the fraction of microblogs as exogenous input [26], denoted as AR(${lag}_{NC}$)+Mblog(${lag}_{Mblog}$):

$y_{t}=\sum_{i=1}^{{lag}_{NC}} a_{i}y_{t-i}+\sum_{h=1}^{{lag}_{Mblog}} c_{h}z_{t-h}+eMI_{t}+f+\varepsilon_{t},$ $\varepsilon_{t} \sim N(0, \sigma^{2})$ (3)

1. Autoregression model adding search volume as exogenous input [36], denoted as AR(${lag}_{NC}$)+Query(${lag}_{Query}$):

$y_{t}=\sum_{i=1}^{{lag}_{NC}} a_{i}y_{t-i}+\sum_{k=1}^{{lag}_{Query}} d_{k}s_{t-k}+eMI_{t}+f+\varepsilon_{t},$ $\varepsilon_{t} \sim N(0, \sigma^{2})$ (4)

1. Multivariable linear model adding the fraction of real-time online news articles, the fraction of microblogs, and search query volume into historical COVID-19 official report data [11,20], denoted as AR(${lag}_{NC}$)+News(1)+Mblog(1)+Query(1):

$y_{t}=\sum_{i=1}^{{lag}_{NC}} a_{i}y_{t-i}+b_{1}x_{t-1}+c_{1}z_{t-1}+d_{1}s_{t-1}+eMI_{t}+f+\varepsilon_{t},$ $\varepsilon_{t} \sim N(0, \sigma^{2})$ (5)

where${lag}_{NC}$is the time lag that leads to the highest forecasting accuracy for the baseline model AR(${lag}_{NC}$), ${lag}_{News}$ is the time lag that leads to the highest forecasting accuracy for model AR(${lag}_{NC}$)+News(${lag}_{News}$), ${lag}_{Mblog}$ is the time lag that leads to the highest forecasting accuracy for model AR(${lag}_{NC}$)+Mblog(${lag}_{Mblog}$), and ${lag}_{Query}$ is the time lag that leads to the highest forecasting accuracy for model AR(${lag}_{NC}$)+News(${lag}_{Query}$) (see Multimedia Appendix 2 for detailed lag selections).
