# Supplementary material for: Enhancing COVID-19 Epidemic Forecasting Accuracy by Combining Real-time and Historical Data From Multiple Internet-Based Sources: Analysis of Social Media Data, Online News Articles, and Search Queries
Source: JMIR Public Health Surveill. 2022 Jun 16;8(6):e35266. doi: 10.2196/35266 (PMC9205424; doi:10.2196/35266)
Supplement: Multimedia Appendix 4 [file publichealth_v8i6e35266_app4.docx]

**Multimedia Appendix 4. Accuracy indexes.**

The RMSE, R2, and MAE of the estimator $\hat{m}$ to the target daily new confirmed COVID-19 related confirmed case counts $m$ are defined, respectively, as RMSE=${[(1/n)\sum_{t=1}^{n} {(\hat{m}_{t}-m_{t})}^{2}]}^{1/2}$, MAE=$(1/n)\sum_{t=1}^{n} |\hat{m}_{t}-m_{t}|$, and MAPE=$\left( \frac{1}{n} \right)\sum_{t=1}^{n} \left| \hat{m}_{t}-m_{t} \right|/m_{t}$. The correlation of estimator $\hat{m}$ to the target daily new confirmed case counts $m$ is their sample correlation coefficient. The correlation of increment between $\hat{m}$ and $m$ is defined as Corr. of increment($\hat{m}_{t}, m_{t}$) = Corr($\hat{m}_{t}-\hat{m}_{t-1}, m_{t}-m_{t-1}$).
