# Supplementary material for: Enhancing COVID-19 Epidemic Forecasting Accuracy by Combining Real-time and Historical Data From Multiple Internet-Based Sources: Analysis of Social Media Data, Online News Articles, and Search Queries
Source: JMIR Public Health Surveill. 2022 Jun 16;8(6):e35266. doi: 10.2196/35266 (PMC9205424; doi:10.2196/35266)
Supplement: Multimedia Appendix 5 [file publichealth_v8i6e35266_app5.docx]

**Multimedia Appendix 5. Collinearity diagnostics.**

To test whether social media data, online news articles, and search queries could serve as independent sources to enhance COVID-19 surveillance, we also conducted collinearity diagnoses in our proposed model (taking both real-time and historical data into account) and a basic model (taking only real-time data into account). The basic model’s formula is below, and denoted as AR($1$)+News(1)+Mblog(1)+Query(1):

$y_{t}=a_{1}y_{t-1}+b_{1}x_{t-1}+c_{1}z_{t-1}+d_{1}s_{t-1}+eMI_{t}+f+\varepsilon_{t},$ $\varepsilon_{t} \sim N(0, \sigma^{2})$ (6)

The collinearity statistics for our proposed and basic models were shown in Table A1, A2, and A3 below. The results showed that the basic model using real-time data from multiple Internet-based data showed a low level of multicollinearity in mainland China except Hubei (VIF for online news articles is 2.822, VIF for microblogs is 3.486, and VIF for search queries is 3.103), and showed small to moderate levels of multicollinearity in Hubei province (VIF for online news articles is 1.982, VIF for microblogs is 4.464, and VIF for search queries is 4.493). Our proposed model using both real-time and historical data showed moderate to severe levels of multicollinearity in Hubei and the rest of mainland China (VIF ranges from 4.831 to 10.877 in mainland China except Hubei; VIF ranges from 5.351 to 44.852 in Hubei province).

The multicollinearity levels for different Internet-based data sources in the basic model combining real-time data are relatively low when tested in Hubei and the rest of mainland China. This implies that real-time social media data, online news articles, and search queries are independent of each other in supplementing COVID-19 surveillance. The Internet-based data sources’ multicollinearity levels range from moderate to severe in our proposed model. This shows that adding the historical data to the forecasting model would cause additional collinearity, which could influence our judgment of single Internet-based data’s influence on the final forecasting result. However, as Kutner et al. stated, “The fact that some or all predictor variables are correlated among themselves does not, in general, inhibit our ability to obtain a good fit nor does it tend to affect inferences about mean responses or predictions of new observations” (p283) [1], multicollinearity affects the coefficients and p-values, but does not influence the precision of the predictions. Adding historical data for a single Internet-based data source has also been widely used in various infectious disease surveillance. As our primary goal is to enhance COVID-19 forecasting accuracy, we argue that multicollinearity doesn’t affect the predictive power of our proposed model. In addition, combining both real-time and historical data into our proposed model improves forecasting accuracy in terms of both magnitude and statistical significance over existing methods.

## **Table A1.** Collinearity Statistics for Basic Model Using Real-time Data Only from Multiple Sources (AR(1)+News(1)+Mblog(1)+Query(1)): Hubei Province and the Rest of Mainland China.

| Independent Variables | Collinearity Statistics for mainland China except Hubei | | Collinearity Statistics for Hubei Province | |
| --- | --- | --- | --- | --- |
|  | Tolerance | VIF | Tolerance | VIF |
| $y_{t-1}$ | 0.196 | 5.096 | 0.640 | 1.563 |
| $x_{t-1}$ | 0.354 | 2.822 | 0.505 | 1.982 |
| $z_{t-1}$ | 0.287 | 3.486 | 0.224 | 4.464 |
| $s_{t-1}$ | 0.322 | 3.103 | 0.223 | 4.493 |

* $y_{t}$ is the logit-transformed COVID-19 new confirmed case proportion at day t, $x_{t}$is the log-transformed fraction of COVID-19 related online news articles at day t, $z_{t}$is the log-transformed fraction of COVID-19 related microblogs at day t, and $s_{t}$is the log-transformed COVID-19 related search volume at day t.

## **Table A2.** Collinearity Statistics for Our Proposed Model Using Real-time and Historical Data from Multiple Sources (AR(7)+News(1)+Mblog(10)+Query(1)): Mainland China except Hubei.

| Independent Variables | Collinearity Statistics | |
| --- | --- | --- |
|  | Tolerance | VIF |
| $y_{t-1}$ | 0.059 | 16.918 |
| $y_{t-2}$ | 0.023 | 43.799 |
| $y_{t-3}$ | 0.022 | 45.638 |
| $y_{t-4}$ | 0.025 | 40.797 |
| $y_{t-5}$ | 0.020 | 49.561 |
| $y_{t-6}$ | 0.017 | 57.262 |
| $y_{t-7}$ | 0.020 | 49.647 |
| $x_{t-1}$ | 0.092 | 10.877 |
| $z_{t-1}$ | 0.180 | 5.550 |
| $z_{t-2}$ | 0.207 | 4.831 |
| $z_{t-3}$ | 0.178 | 5.618 |
| $z_{t-4}$ | 0.164 | 6.108 |
| $z_{t-5}$ | 0.133 | 7.496 |
| $z_{t-6}$ | 0.162 | 6.167 |
| $z_{t-7}$ | 0.175 | 5.710 |
| $z_{t-8}$ | 0.145 | 6.897 |
| $z_{t-9}$ | 0.110 | 9.124 |
| $z_{t-10}$ | 0.138 | 7.223 |
| $s_{t-1}$ | 0.101 | 9.912 |

* $y_{t}$ is the logit-transformed COVID-19 new confirmed case proportion at day t, $x_{t}$is the log-transformed fraction of COVID-19 related online news articles at day t, $z_{t}$is the log-transformed fraction of COVID-19 related microblogs at day t, and $s_{t}$is the log-transformed COVID-19 related search volume at day t.

## **Table A3.** Collinearity Statistics for Our Proposed Model Using Real-time and Historical Data from Multiple Sources (AR(1)+News(3)+Mblog(1)+Query(3)): Hubei Province.

| Independent Variables | Collinearity Statistics | |
| --- | --- | --- |
|  | Tolerance | VIF |
| $y_{t-1}$ | 0.369 | 2.713 |
| $x_{t-1}$ | 0.161 | 6.202 |
| $x_{t-2}$ | 0.066 | 15.129 |
| $x_{t-3}$ | 0.094 | 10.599 |
| $z_{t-1}$ | 0.187 | 5.351 |
| $s_{t-1}$ | 0.031 | 32.584 |
| $s_{t-2}$ | 0.022 | 44.852 |
| $s_{t-3}$ | 0.032 | 30.891 |

* $y_{t}$ is the logit-transformed COVID-19 new confirmed case proportion at day t, $x_{t}$is the log-transformed fraction of COVID-19 related online news articles at day t, $z_{t}$is the log-transformed fraction of COVID-19 related microblogs at day t, and $s_{t}$is the log-transformed COVID-19 related search volume at day t.

## **Reference**

1. Kutner M, Nachtsheim C, Neter J, Li W. Applied Linear Statistical Models. 5th International Edition ed.
